# Developing a Reading Concentration Monitoring System by Applying an Artificial Bee Colony Algorithm to E-Books in an Intelligent Classroom

**DOI:** 10.3390/s121014158

**Published:** 2012-10-22

**Authors:** Chia-Cheng Hsu, Hsin-Chin Chen, Yen-Ning Su, Kuo-Kuang Huang, Yueh-Min Huang

**Affiliations:** 1 Department of Engineering Science, National Cheng Kung University, No.1, University Road, Tainan City 701, Taiwan; E-Mails: chia.cheng.hsu.tw@gmail.com (C.-C.H.); luckyqq50@gmail.com (H.-C.C.); yenning@mail.tn.edu.tw (Y.-N.S.); 2 Department of Information Management, National Penghu University of Science and Technology, No.300, Liuhe Road, Magong City, Penghu County 880, Taiwan; E-Mail: kkhuang@npu.edu.tw

**Keywords:** reading concentration, sensor technology, artificial bee colony algorithm, e-books, intelligent classroom

## Abstract

A growing number of educational studies apply sensors to improve student learning in real classroom settings. However, how can sensors be integrated into classrooms to help instructors find out students' reading concentration rates and thus better increase learning effectiveness? The aim of the current study was to develop a reading concentration monitoring system for use with e-books in an intelligent classroom and to help instructors find out the students' reading concentration rates. The proposed system uses three types of sensor technologies, namely a webcam, heartbeat sensor, and blood oxygen sensor to detect the learning behaviors of students by capturing various physiological signals. An artificial bee colony (ABC) optimization approach is applied to the data gathered from these sensors to help instructors understand their students' reading concentration rates in a classroom learning environment. The results show that the use of the ABC algorithm in the proposed system can effectively obtain near-optimal solutions. The system has a user-friendly graphical interface, making it easy for instructors to clearly understand the reading status of their students.

## 1. Introduction

In recent years, the use in educational contexts of e-books, which are learning materials in electronic form, has attracted more attention [1–3]. However, researchers and educators not only have to understand the effect on student learning that arise from using e-books, but also have to know the learning status of students in the classroom when reading e-books, such as reading concentration rates, emotions, and anxiety levels. With the development of computer networks, sensor technologies have been widely applied in various fields, such as biomedical engineering [4,5], material science [6,7], navigation [8–11], and warehousing [12–14]. In addition, more and more educators have applied sensor technologies to classroom activities and in learning environments, because they have the advantages of low cost and fast data collection. Some related applications of sensor technologies in education include context aware ubiquitous learning [15–21], augmented reality [22–26], e-books [27], intelligent classrooms [28,29], and the physiological measurement of students [30,31]. The results of these previous studies show that using sensor technologies can promote the learning achievement and motivation of students in their learning environments.

Sensors make measurements and can send the related data to various devices. With regard to physical measurements, sensors can be used to monitor pressure, temperature, humidity, oxygen, and gravity, many of which have been applied in learning environments. Hwang *et al.* proposed a set of basic criteria and strategies that can be used for context-aware ubiquitous learning [32], and used radio-frequency identification (RFID) tags with PDAs to increase the botanical knowledge of students. Wang and Wu proposed an adaptive u-learning system to improve learning effectiveness using context-aware technology and recommendation algorithms [20]. In addition, Chen and Huang presented a context-aware ubiquitous learning system (CAULS) based on RFID, wireless networks, embedded handheld devices, and database technologies to detect and examine the real-world learning behaviors of students [33]. The findings obtained by Chen and Huang suggested that this innovative approach enhanced the students' learning intentions. In a science course, Hwang *et al.* proposed a context-aware u-learning environment that was developed for guiding inexperienced researchers to practice single-crystal X-ray diffraction operations. The results of their study showed that the benefits of this innovative approach are: (a) it is systematic, authentic, and economical, and (b) this context-aware technology could be applied to help students understand and carry out complex science experiments in different domain, such as physics, chemistry or biotechnology [19]. Sayed *et al.* presented an augmented reality student card (ARSC) as an application of augmented reality (AR) in an educational context. In their work, the ARSC was used by a number of students of both genders, aged between 10 and 17, and it was widely accepted by students [24]. Huang *et al.* developed an interactive e-book learning system (IELS) for elementary school students, and concluded that its touch screen-based learning tracking technique was able to provide a clear view of the students' learning processes [27].

Researchers have also used sensor technologies to obtain students' physiological data. For example, Wu *et al.* proposed the concept of an intelligent classroom, and they presented a prototype system that integrated various sensors and networking technologies to develop an effective e-book learning environment for students [34]. Su *et al.* proposed the simple system of learning concentration detection based on a webcam and pressure cushion [31]. Hwang *et al.* also developed a sensor-assisted learning system (SALS) based on various sensor technologies, including a pressure sensor, heartbeat sensor, and webcam. Their study used decision trees (C4.5 Algorithm) to build the structure of the SALS, and it was found that recognition accuracy with regard to the students' concentration levels ranged from 86% to 90% [30]. Previous studies [20,30–32,34] demonstrate that sensor technologies have now become an important tool for use in education, and thus the current work aims to use sensors to help instructors discover students' reading concentration rate and to raise their learning effectiveness.

Reading concentration measures how actively a student actively pays attention to the learning materials and contents during the learning process. In the past few decades, there were several terms proposed for concentration on learning, and one of them is attention. Attention refers to the awareness [35]. Attention is the ability to focus or sustain on an action without interference from external stimuli. Davenport and Beck [36] stated that attention is the ability to focus on specific items, while Dumont [37] pointed out that focused attention is the same as concentration. Jensen [38] stated that attention in an educational environment usually refers to an externally focused concentration. Concentration or attention plays an important role in improved learning [39,40], as students must focus on the learning materials, and then continue to do so for some time if they are to retain the information they contain [41,42]. Corno [43] pointed out that enhances learning outcomes are often seem for students with high concentration or motivation and student concentration is a key factor in the effectiveness of instructors' teaching strategies [44].

In general, understanding the individual learning status of each student can help instructors develop more appropriate teaching strategies and enhance the quality of learning that occurs in the classroom. However, in traditional classroom instructors usually need to teach many students at once, and thus it is difficult for instructors to pay attention to the learning status of each student to give them appropriate assistance. In the current study, reading concentration refers to the attention that is focused on reading or learning. Delgado *et al.* indicated that students' learning concentration is a crucial factor in their learning performance [45], and while concentration can be easily observed from the students' behavior [46], it is hard to analyze using technology. Therefore, some studies have tried to examine learning concentration by using sensor technologies, and the results indicate that this approach can be effective [30,31]. In addition, by using sensor technologies instructors can understand some of the crucial factors that reveal learning concentration, including facial expressions, and eye and body movements.

Monitoring learning behavior by using sensor technologies in an educational environment is a challenging exercise, especially when various different sensors are used, and the data needs to be combined. Over the past few years, several studies have used an artificial bee colony algorithm (ABC) to solve different combinatorial problems [47], and Karaboga [48] indicated that the performance of the ABC is better than, or at least similar to, that of an evolutionary strategy (ES) or particle swarm optimization algorithm (PSO).

In recent years the Taiwanese government has conducted a series of e-learning experimental projects [49], examining the use of intelligent classrooms, e-books, and digital Chinese learning materials. An intelligent classroom is defined as a classroom that integrates of information technology, such as interactive whiteboards, e-books, and sensors, to facilitate the teacher teaching and enhance student learning. One of the aims when using digital learning materials is to monitor reading concentration. However, it is difficult for instructors to observe the learning of individual students so that they can provide enough attention to help their learning.

The purpose of this study was to develop a reading concentration monitoring system using sensor technologies that includes a webcam, heartbeat sensor, and blood oxygen sensor to help instructors observe the learning status of students when they are reading digital materials in an intelligent classroom. Using the three types of sensors, the proposed system can evaluate the reading concentration of students. Moreover, the ABC algorithm is used to search for the students that are not paying attention to the digital learning materials. The reading concentration data can help instructors understand the learning status of the individual students, and thus adopt more suitable teaching strategies based on this.

## 2. Artificial Bee Colony (ABC) Algorithm

The artificial bee colony (ABC) algorithm was first proposed by Karagoga to search for near-optimal solutions [47]. The ABC algorithm is based on the foraging behaviors of bees, and mimics the interactions that occur in swarm intelligence to solve optimization problems [48]. Swarm intelligence, like particle swarm optimization, has been successfully applied in various fields, such as wireless sensor networks, e-learning, flow shop problems, scheduling problems, and clustering problems [50–59].

**Figure 1.**
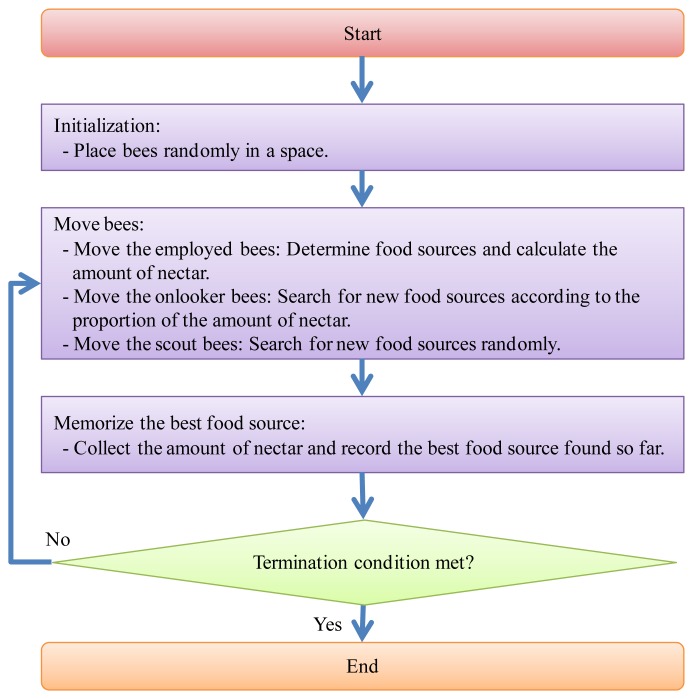
Flow chart of the ABC algorithm.

In the real environment, a social community of bees in a colony is composed of three main types of bees, which are a queen as the kernel, a few drones for reproducing the next generation, and a large number of workers that look for pollen and take care of larvae in the colony. The ABC algorithm is based on the behavior of worker bees, which it divides into employed, onlooker, and scout bees. Scout bees are responsible for searching for new food sources and reporting the amount of nectar at each location. After the scout bees have gathered this information, the employed bees fly to the neighborhood of the food sources to search for the new source and find out how much nectar they have. The onlooker bees wait for information about the food sources from the employed bees, and then use this to go out and gather nectar. Figure 1 shows the detailed procedure of the ABC algorithm.

At first, assume that bees are generated in the initialization step. Half of the *N* bees are selected to randomly spread out to search for food resources in the solution space. Each bee selects a position and retains the amount of nectar there in its memory, with the results of this being used to produce the first fitness value. Next, each employed bee flies to the selected food source and chooses a new position near the original ones. After comparing the amount of nectar at both food sources, the employed bee selects the one with the most nectar as the new food source. Third, the onlooker bees stay at the hive to wait for information about the amount of nectar at the selected food sources. The onlooker bees then select a food source, and the probability that a particular food source will be selected increases along with the amount of nectar that it has.

The probability of a food source will be selected is shown in the following formula:

(1) $$P_{i}=\frac{F\left(\theta_{i}\right)}{\sum_{k=1}^{E}F\left(\theta_{k}\right)}$$

where *θ_i_* is the position of the food source *i*, *F*(*θ_i_*) means the amount of nectar at the *i*th food source, *F*(*θ_i_*) means the amount of nectar at the *k*th food source, and *E* is the number of employed bees. After moving to the selected food source, each onlooker bee selects a position near the original food source using the following equation, and obtains the amount of nectar:

(2) $$x_{id}\left(t+1\right)=\theta_{id}+\phi \left(\theta_{id}\left(t\right)-\theta_{kd}\left(t\right)\right)$$

where *t* indicates the number of iterations, *i* is the number of the employed bee, *d* is the dimension of the position, *k* is the randomly chosen employed bee, *θ_id_* means the position of dimension *d* of the *i*th onlooker bee, *θ_kd_* means the position of dimension *d* of the randomly chosen employed bee, and *φ*() is a random value in the range [−1, 1], while 1 ≤ *i* ≤ *E*, 1 ≤ *d* ≤ *D* and 1 ≤ *k* ≤ *E*. Fourth, scout bees are used to randomly search for new food sources. In the ABC algorithm, each food source selected by the employed bee has a parameter to record the number of the food source selected. Once the parameter value exceeds the predetermined number of iterations, known as the limit, the food source is abandoned. The employed bee becomes a scout bee and searches for a new food source to replace the original one. The operation of the scout bee is as follows:

(3) $$\theta_{id}=\theta_{id_{\min}}+\eta \times \left(\theta_{id_{\max}}-\theta_{id_{\min}}\right)$$

where *η* is a random value in the range [0, 1]. The scout bee then becomes an employed bee again. Finally, after the bees complete a search process, the best fitness value is acquired through a comparison of all the fitness values. At the end of each round, the algorithm checks whether the bees should continue to search for new food sources or not. If the termination condition is satisfied, the algorithm stops the search process and then outputs the related results of the fitness values and the food sources.

## 3. Reading Concentration Monitoring System Scheme

This study presents a reading concentration monitoring system as a method of observing the learning status of students and providing the results to the instructors, so that they can improve learning outcomes. To determinate the students' learning status, the system uses three types of sensor technologies, namely a webcam, heartbeat sensor, and blood oxygen sensor (fingertip oximeter), in order to gather eye gaze, heartbeat, and blood oxygen saturation data. The important notations and variables used in this paper are listed in Table 1.

The reading concentration monitoring system can acquire the learning status of each student in an intelligent classroom, and then record their concentration rates in the database of this system. The instructor can then use the results to modify the learning materials or teaching strategies. Assume that the reading concentration monitoring system observes *N* students in a teaching environment, who are denoted as *L*_1_, *L*_2_, … *L_N_*. The learning behaviors in the activity are monitored by sensors and recorded in the database. There are *M* students, which are a subset of *N*, that are selected from the database for learning status to monitor their learning status. The term *s_i_* indicates whether a student is selected or not. Based on the three variables obtained from the sensors, the system can reveal whether the students are attentive or not.

First, networked webcams are used to observe the positions of students' eyes. Hwang *et al.* proposed the SALS that captured the image of an individual student by a webcam, and analyzed the image whether the eyes were in the image for identifying the student's attention [30]. According to Hwang *et al.*, networked webcams in our method are used to observe the positions of students' eyes and calculate the center position of their two eyes to determine their reading fixation. This study determines that the student pays attention in reading materials presented on the screen for acquiring knowledge, when the center position of the student's eyes is in the image. On the other hand, the networked webcam captures the eyes' center point *P_i_* of student *i* on the screen *T* times during a fixed period for detecting reading fixation. The center point is composed of a two-dimensional vector [*p_i,j_*,_1_ *p_i,j_*,_2_], which includes the x-axis and the y-axis dimensions, where *j* means the *j*-th measurement. Therefore, the definition of center point *P_i_* is as follows:

(4) $$P_{i}=\left[\begin{array}{cc} p_{i,1,1} & p_{i,1,2}\\ p_{i,2,1} & p_{i,2,2}\\ p_{i,3,1} & p_{i,3,2}\\ \cdots & \cdots \\ p_{i,T,1} & p_{i,T,2} \end{array}\right]$$

The element *p_i,j,k_* of matrix *P_i_* represents that student *i* looks at point *k* at a time point *j*, where 1 ≤ *i* ≤ *N*, 1 ≤ *j* ≤ *T*, and 1 ≤ *k* ≤ 2. In order to determine whether student *i* is paying attention to the screen, a matrix *G_i_* = [*g_i_*_,1_ *g_i_*_,2_ *g_i_*_,3_ ⋯ *g_i,T_*]*^Tr^* is used to set the value of reading fixation. The element *g_i_*,*_j_* of matrix *G_i_* represents the degree of reading fixation. *g_i_*,*_j_* is set to 1 if one *p_i_*,*_j_*,*_k_* exists in the position of the screen, and 0 otherwise. The definition of the reading fixation rate *r_i_* is used to obtain the level of fixation of student *i*, as follows:

(5) $$r_{i}=\frac{\overset{T}{\underset{j=1}{\text{∑}}}g_{i,j}}{T}$$

The value of reading fixation rate *r_i_* of student *i* increases, as the reading fixation of student *i* on the screen increases. The *μ* represents the reading fixation rate of the selected students with regard to the reading materials. Therefore, the strength of relationship *μ* between the reading fixation rate and the concentration of the students is presented as follows:

(6) $$\mu =\frac{\overset{N}{\underset{i=1}{\text{∑}}}\left(s_{i}\times \left(\frac{\overset{T}{\underset{j=1}{\text{∑}}}g_{i,j}}{T}\right)\right)}{\max\left(\overset{N}{\underset{i=1}{\text{∑}}}\left(s_{i}\right),\sigma \right)}$$

Second, heartbeat detectors are used to observe the heartbeat rates of the students. Previous studies found that there are significant correlations between attention and heartbeat rate, which goes down when students pay more attention to learning materials, and *vice versa* [60–62]. Hwang *et al.* thus proposed the SALS, which obtained the heartbeat rates of an individual student via a heartbeat sensor, and analyzed these to assess their level of attention [30]. In our work the system automatically detects the heartbeat of student *i* for *T* times during a fixed period via the heartbeat detector. With regard to heartbeats of students, both the heart rate and its variation are considered. When a student concentrates on reading materials, their heart rate is at a certain value, and the variation is relatively low. The target heart rate *h_i,Target_* is the measured heart rate, when student *i* concentrates on reading materials. The heart rate of student *i* is presented as the matrix *H_i_* = [*h_i_*,_1_ *h_i_*,_2_ *h_i_*,_3_ …*h_i,T_*]*^Tr^*, and the element *h_i,j_* means the heart rate measured at a fixed point of time *j*. The average heart rate *a_i_* is presented as follows:

(7) $$a_{i}=\frac{\overset{T}{\underset{j=1}{\text{∑}}}h_{i,j}}{T}$$

To determine the slope, the system uses the variation of heart rate Δ*b_i_* of student *i*:

(8) $$\Delta b_{i}=\{\begin{array}{ll} 0, & \text{if}\;h_{i,T}<h_{i,1}-h_{L}\\ \frac{1}{2}, & \text{if}\;h_{i,T}\geq \left(h_{i,1}-h_{L}\right)\;\text{and}\;h_{i,T}\leq \left(h_{i,1}+h_{U}\right)\\ 1, & \text{if}\;h_{i,T}>h_{i,1}+h_{U} \end{array}$$

The variable λ represents the heartbeat status of the selected students. When the students have a high level of reading concentration, the value of *λ* is also high. The strength of relationship *λ* between heart rate and the students' concentration is showed as follows:

(9) $$\lambda =\frac{\overset{N}{\underset{i=1}{\text{∑}}}\left(s_{i}\times \left(\left(1-\beta \right)\times e^{-0.01\times {\left(a_{i}-h_{i,\text{Target}}\right)}^{2}}+\beta \times \Delta b_{i}\right)\right)}{\max\left(\overset{N}{\underset{i=1}{\text{∑}}}\left(s_{i}\right),\tau \right)}$$

Finally, blood oxygen detectors are used to observe the blood oxygenation of the students. In general, a learner in the state of hypoxia is more likely to experience memory loss, lack of concentration, lethargy, and mental depression. There are various methods for measuring blood oxygen saturation such as saturation of peripheral oxygen (SpO_2_), venous oxygen saturation (SvO_2_), and arterial oxygen saturation (SaO_2_). Saturation of peripheral oxygen (SpO_2_) is a nonintrusive measurement, and has been used to estimate mental and physical fatigue [63]. In our work the system automatically detects the oxygenation of the blood via a physical SpO_2_ detector. In general, when the oxygen in the blood is lower, the student is more easily fatigued and is less likely to concentrate on the reading learning materials. Two factors are considered here, the oxygen in the blood and the variation of this. The oxygen in the blood is presented as the matrix *O_i_* = [*o_i_*,_1_ *o_i_*,_2_ *o_i_*,_3_ …*o_i,T_*]*^Tr^*,. The element *o_i,j_* of matrix *O_i_* is the oxygen in the blood for student *i* at a time point *j*. In order to find students that are within a specific range, such as the range from 100% to 90%, the system determines the matrix *X_i_* = [*x_i_*,_1_ *x_i_*,_2_ *x_i_*,_3_ …*x_i,T_*]*^Tr^* to transform original oxygen of blood, and the element *x_i,j_* of matrix *X_i_* is presented as follows:

(10) $$x_{i,j}=\{\begin{array}{cc} 0, & {\text{if o}}_{i,j}<o_{i,\text{Threshold}}\\ \frac{o_{i,j}-o_{i,\text{Threshold}}}{o_{i,\max}-o_{i,\min}}, & {\text{if o}}_{i,j}\geq o_{i,\text{Threshold}} \end{array}$$

The average transformed oxygen *x_i_* in the blood during a time is presented as follows:

(11) $$x_{i}=\frac{\overset{T}{\underset{j=1}{\text{∑}}}x_{i,j}}{T}$$

In order to determine the slope, the system uses the variation of oxygen in the blood Δ*y_i_*:

(12) $$\Delta y_{i}=\{\begin{array}{ll} 0, & \text{if}\;o_{i,j}<o_{i,\text{Threshold}}\\ 0, & \text{if}\;o_{i,j}\geq o_{i,\text{Threshold}},\;\text{and}\;x_{i,T}<\left(x_{i,1}-x_{L}\right)\\ \frac{1}{2}, & \text{if}\;o_{i,j}\geq o_{i,\text{Threshold}},x_{i,T}\geq \left(x_{i,1}-x_{L}\right),\;\text{and}\;x_{i,T}\leq \left(x_{i,1}+x_{U}\right)\\ 1, & \text{if}\;o_{i,j}\geq o_{i,\text{Threshold}},\;\text{and}\;x_{i,T}\geq \left(x_{i,1}-x_{U}\right) \end{array}$$

The variable *π* represents the oxygen situation in blood of the selected students with regard to the reading materials. When the selected students have higher levels of oxygen in the blood, the value of *π* is higher. The strength of relationship *π* between the oxygen in the blood and the concentration of the students is shown as follows:

(13) $$\pi =\frac{\overset{N}{\underset{i=1}{\text{∑}}}\left(s_{i}\times \left((1-ɛ)\times \frac{\overset{T}{\underset{j=1}{\text{∑}}}x_{i,j}}{T}+ɛ\times \Delta y_{i}\right)\right)}{\max\left(\overset{N}{\underset{i=1}{\text{∑}}}\left(s_{i}\right),\omega \right)}$$

The fitness function *F*(*s*) in the reading concentration monitoring system model consists of the following three constraints. The fitness value is obtained by the fitness function *F*(*s*), and the fitness value is updated by a process of computation and iteration to find students who are not paying attention. When the students have a low level of reading concentration, their presented fitness value will be lower than that of the other students with more reading concentration. If the selected students have a minimal fitness value, the system will select the optimal solution. The formal definition of the system model is as follows:

(14) $$F\left(s\right)=\frac{\overset{N}{\underset{i=1}{\text{∑}}}\left(s_{i}\times \left(\frac{\overset{T}{\underset{j=1}{\text{∑}}}g_{i,j}}{T}\right)\right)}{\max\left(\overset{N}{\underset{i=1}{\text{∑}}}\left(s_{i}\right),\sigma \right)}+\frac{\overset{N}{\underset{i=1}{\text{∑}}}\left(s_{i}\times \left(\left(1-\beta \right)\times e^{-0.01\times {\left(a_{i}-h_{i,\text{Target}}\right)}^{2}}+\beta \times \Delta b_{i}\right)\right)}{\max\left(\overset{N}{\underset{i=1}{\text{∑}}}\left(s_{i}\right),\tau \right)}+\frac{\overset{N}{\underset{i=1}{\text{∑}}}\left(s_{i}\times \left((1-ɛ)\times \frac{\overset{T}{\underset{j=1}{\text{∑}}}x_{i,j}}{T}+ɛ\times \Delta y_{i}\right)\right)}{\max\left(\overset{N}{\underset{i=1}{\text{∑}}}\left(s_{i}\right),\omega \right)}$$

## 4. Framework of a Reading Concentration Monitoring System

In this section, we present a reading concentration monitoring system that collects learning behaviors through sensor technologies and then finds the students' actual reading concentration rates in an actual learning environment. The related procedures are described in detail.

### 4.1. Architecture

**Figure 2.**
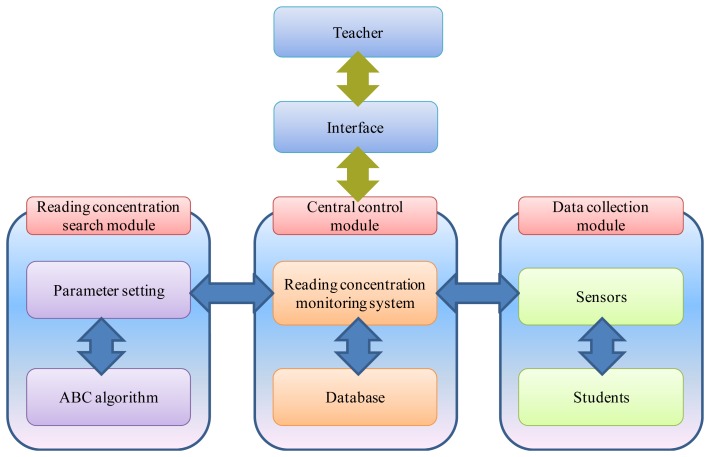
Framework of the reading concentration monitoring system.

Figure 2 shows the architecture of the reading concentration monitoring system. There are three main components, which are the central control module, data collection module, and reading concentration search module.

1. Central control module: The central control module is the main module of the system, and is responsible for information exchange. This module receives the reading behaviors of students and records them into the database. It then transforms these behaviors into reading concentration data using an equation. The results are then provided to the reading concentration search module for searching purposes.
2. Data collection module: The main purpose of the data collection module is to observe the reading behaviors of students, and this is carried out using three types of sensors, which are a webcam, heartbeat sensor, and blood oxygen sensor. The heartbeat and blood oxygen sensors are embedded on the side of the computer mouse. The webcam captures the center point of the student's eyes on the screen. The heartbeat sensor detects the heartbeat and the variations in this that occur when the student is on reading. Finally, the blood oxygen sensor is used to measure the level of oxygen in the student's blood. The data collected from these sensors is then sent to the central control module.
3. Reading concentration search module: The reading concentration search module is used so that the instructor can find specific information. Via the user interface, the instructor can set specific parameters, such as time and activities, as well the range of view on the screen, variations on heart rate, and the threshold of the oxygen in the blood. After the instructor has set these parameters, the ABC algorithm searches for students that are not paying attention, with the results being sent to the central control module and then presented on the user interface.

### 4.2. Procedure

**Figure 3.**
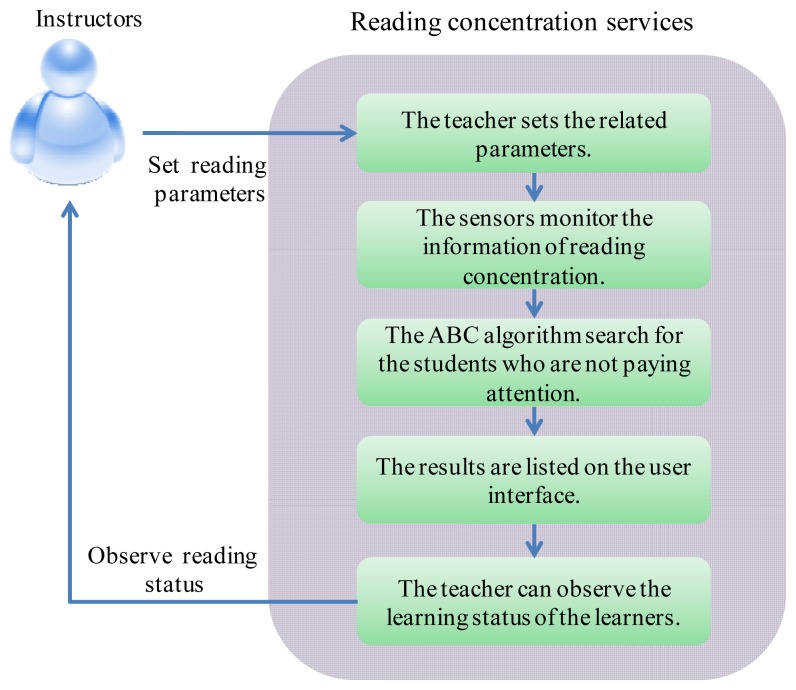
Procedure of the reading concentration monitoring system.

By using the reading concentration system, an instructor can easily understand the learning status of students and the learning atmosphere in class. The flow path of the reading concentration system is shown in the following steps in Figure 3.

#### Step 1

**Figure 4.**
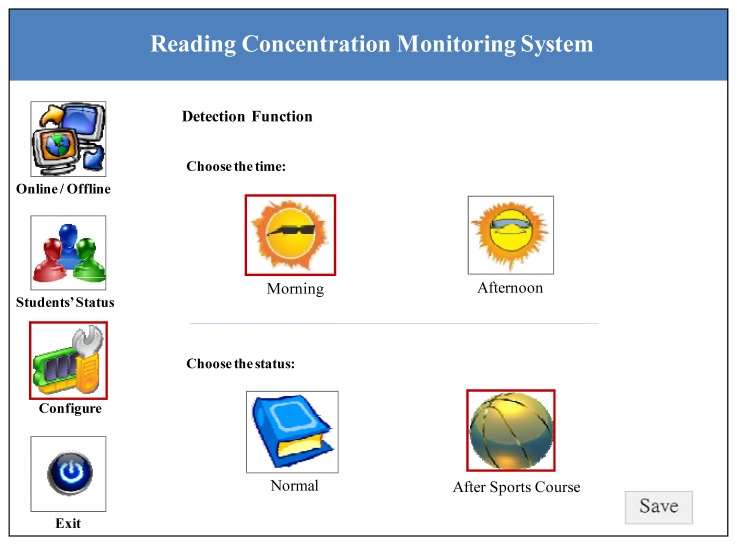
Detection function of the reading concentration monitoring system.

In this system, instructors can set various parameters to suit the specific classroom situation. For example, different times and activities may affect the data, with the former being divided into morning and afternoon, and the latter into a general learning class and a sports class having taken place before the focal class. In general, people have better spirits in the morning than in the afternoon due to variations in blood oxygen. More, the heart rate of is faster after exercise, and this will affect the data that is gathered. Because physiological conditions are affected by many external factors, instructors can select the appropriate detection functions to best monitor the reading concentration of students. Figure 4 shows a screenshot for setting the parameters of the reading concentration monitoring system.

#### Step 2

**Figure 5.**
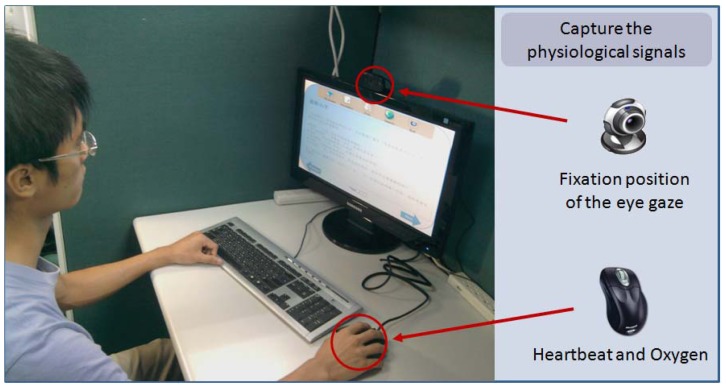
A student using the proposed system.

The sensors monitor various physiological signals while the students are reading the learning materials. Figure 5 shows an example of a student using the proposed system. In this figure, the student uses a PC to read the digital learning materials contained in a software type e-book, and the computer is equipped with the reading concentration monitoring system. The proposed system provides information of reading concentration to help instructors understand the learning status of the individual students and it is used to provide suitable teaching strategies based on the level of students' reading concentration. The assisted teaching features of this proposed system accords with the concept of assisting tools in intelligent classroom.

#### Step 3

The ABC algorithm searches for the students with a low level of attention, based on the data that the sensors collect than the ABC algorithm.

#### Step 4

**Figure 6.**
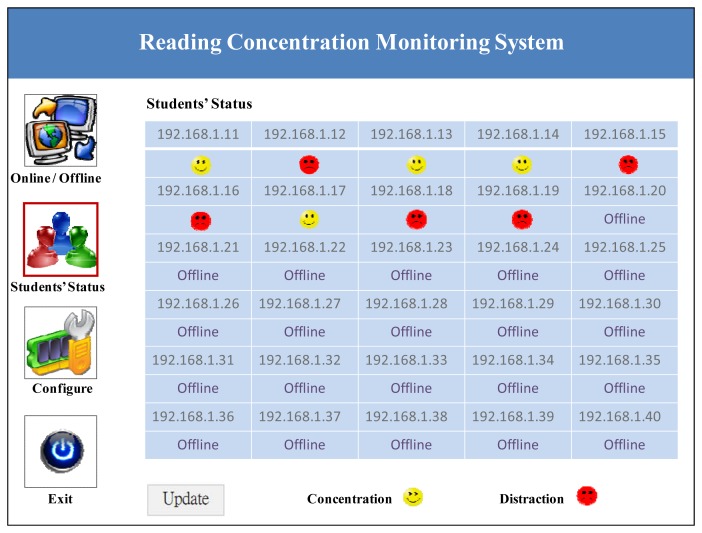
Results of the reading concentration monitoring system.

The results are presented on the user interface, as shown in Figure 6.

#### Step 5

**Figure 7.**
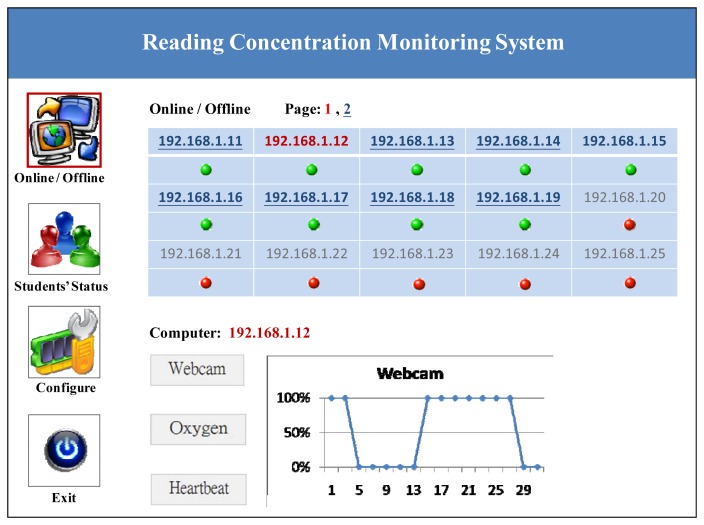
Reading status of an individual student.

The instructor can observe the learning status of the students, and if he or she wants to see more details, these can be obtained by clicking on the students who are not concentrating on the learning materials. Figure 7 shows the reading status of an individual student.

## 5. Experimental Results

To evaluate the performance of the reading concentration monitoring system proposed in this work, a series of experiments were conducted to compare the average fitness values and computation time values. Three main scenarios that were considered as follows: (1) the ABC algorithm and the random search method, (2) 2,000 iterations and different numbers of bees, and (3) 20 bees and different numbers of students. A personal computer which included an Intel Core 2 Quad 2.4 GHz CPU and one 2 GB of RAM was used to run all the experiments. The database included ten datasets of students and the number of students ranged from 150 to 1,500. Every dataset was run ten times each for the ABC algorithm and the random search method. The number of bees was set from 20 to 100. The number of computing iterations was set at 2,000 and the *limit* value of the ABC algorithm was set at 100.

In the first scenario, the random search method and the ABC algorithm were adopted to compare the fitness values and execution time values, as shown in Tables 2 and 3. The results show that the fitness value obtained from the random search method was around 0.9 in ten datasets. The fitness value obtained from the ABC algorithm was under 0.6 at 1,000 iterations, and the fitness value from the algorithm at 2,000 iterations was under 0.3. The maximum execution time of the random search method was under 1 s, while that of the ABC algorithm was about 36 s. As stated above, although the execution time of the ABC algorithm in each dataset was more than that of the random search method, the fitness value of the ABC algorithm is lower than that of the random search method.

**Figure 8.**
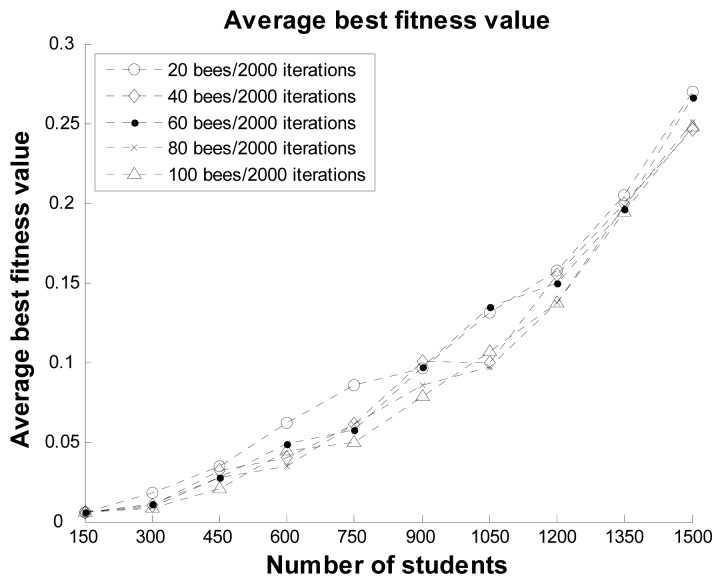
Average best fitness values with 2,000 iterations and different numbers of bees.

**Figure 9.**
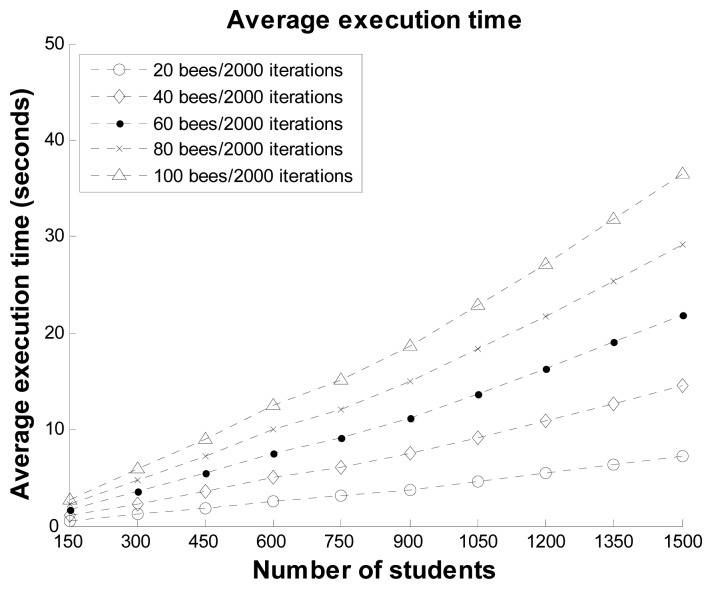
Average execution time values with 2,000 iterations and different numbers of bees.

In the second scenario, the experiment considered the impact of the number of bees on the ABC optimal method for the proposed system. Twenty, 40, 60, 80, and 100 bees were used to compare the fitness values and execution times, as shown in Figures 8 and 9, respectively. It can be observed that the maximum difference in the fitness values among the different numbers of bees was about 0.05 for 750 students. In the other datasets, the difference in the fitness values among the different numbers of bees was also very small. The execution time in each dataset increased almost linearly with the number of bees. The results demonstrate that while a greater number of bees can reduce the best fitness value, the influence on the optimal solutions is small. Therefore, we suggest that the proposed system can use 20 bees in practical applications.

**Figure 10.**
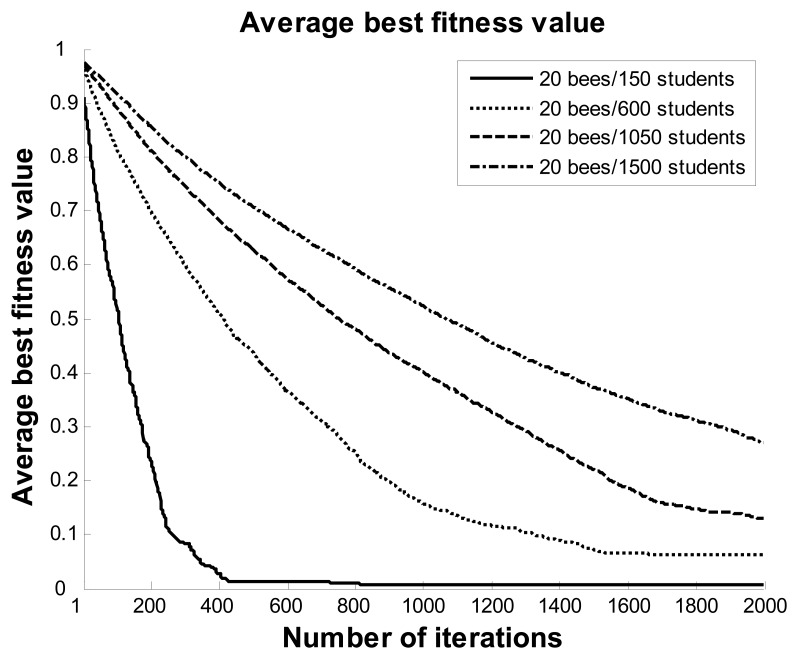
Average best fitness values with 20 bees and different numbers of students.

**Figure 11.**
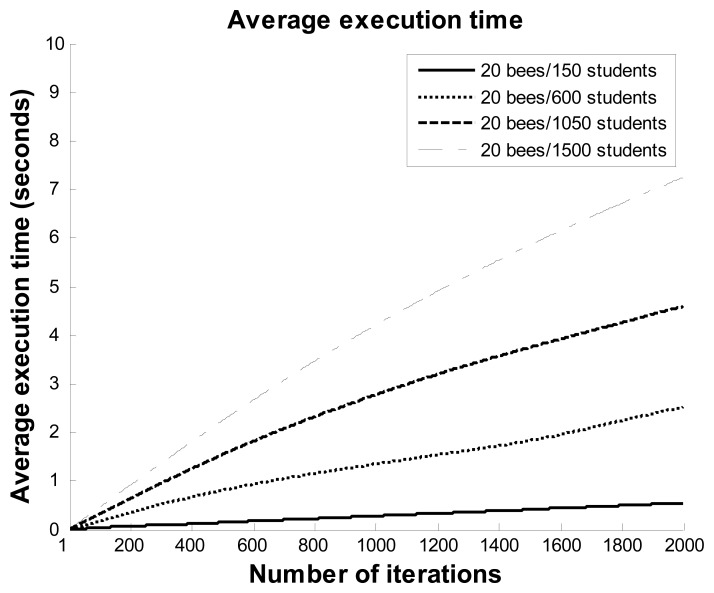
Average execution time values with 20 bees and different numbers of students.

In order to observe the near-optimal solutions of the ABC algorithm in different datasets, four datasets that included 150, 600, 1,050, and 1,500 students were selected for this experiment in the third scenario. Figures 10 and 11 show the best fitness and execution time values of the ABC algorithm with 20 bees and different numbers of students. When the number of students was 400, the fitness value rapidly fell to under 0.1 before about 210 iterations, and then continued to decline to close to zero at 420 iterations. The line for 600 students had a similar falling trend as the line for 150 students. For the lines of 1,050 and 1,500 students, the fitness values decreased following a smooth curve with the number of iterations, and the fitness values fell to less than 0.3 at 2,000 iterations. The maximum execution time for this experiment was under eight seconds. The results show that the ABC algorithm can help users to quickly obtain near-optimal solutions within a reasonable period of time.

## 6. Conclusions

This paper proposed a reading concentration monitoring system using sensor technologies to detect the learning behaviors of students when reading an e-book, based on physiological signals and an artificial bee colony algorithm (ABC). To evaluate the performance of this system in a learning environment, a series of experiments were conducted on ten datasets. The results show that the ABC algorithm can effectively reduce the fitness value, while the random search method may not achieve the near-optimal solution. In addition, the downward trend of the fitness value of the ABC algorithm toward the best solution is very rapid, and has the capability to explicitly produce effective results within a reasonable time to meet the immediate needs of instructors. Based on our findings, the proposed system can help instructors develop more appropriate teaching strategies that are better able to promote student learning motivation, class management, and peer discussions, by understanding the individual learning status of each student. In our future work, we plan to extend this research by using the proposed system in an actual teaching and learning environment, and to assess its practicality based on feedback from instructors.

## Figures and Tables

**Table 1.** Notations and variables used in the fitness function.

**Notation and variable**	**Meaning and explanation**
*s_i_*	The selection of the student 1 ≤ *i* ≤ *N*.
*P_i_*	The matrix, the information of center position of the eyes' point of student *i* on the screen during a certain time.
*p_i,j,k_*	The element of matrix *P_i_*, the position information *k* of center position of the eyes' point of student *i* on the screen at time point *j*.
*G_i_*	The matrix, the information about the reading fixation of the student *i* on a screen during a certain time.
*g_i,j_*	The element of matrix *G_i_*, the degree of the reading fixation of student *i* on the screen on a time point *j*.
*r_i_*	The reading fixation rate of student *i*.
*μ*	The strength of the relationship between the level of reading fixation and the concentration of the students.
*H_i_*	The matrix, the heart rate of student *i* during a time.
*h*_i,j_	The element of matrix *H_i_*, the heart rate of student *i* at a time point *j*.
*a_i_*	The average heart rate of student *i*.
Δ*b_i_*	The variation of the heart rate of student *i*.
*λ*	The strength of the relationship between the heart rate and the concentration of the students.
*O_i_*	The matrix, the blood oxygen for student *i* during a certain time.
*o_i,j_*	The element of matrix *O_i_*, the blood oxygen for student *i* at a time point *j*.
*X_i_*	The matrix, the transformed blood oxygen for the student *i* during a certain time.
*x_i,j_*	The element of matrix *X_i_*, the transformed blood oxygen for student *i* at a time point *j*.
Δ*y_i_*	The variation of blood oxygen for student *i* during a certain time.
*π*	The strength of the relationship between blood oxygen and the concentration of the students.
*F*(*s*)	The fitness function used for the reading concentration monitoring system.

**Table 2.** Comparison of average best fitness values for the random search method and the ABC algorithm.

**Number of students**	**Random search method**	**ABC algorithm (number of bees/number of iterations)**			
		20/1,000	20/2,000	100/1,000	100/2,000
150	0.8905	0.0062	0.0060	0.0060	0.0060
300	0.8977	0.0327	0.0179	0.0098	0.0079
450	0.8880	0.0932	0.0343	0.0471	0.0208
600	0.9063	0.1573	0.0620	0.1137	0.0441
750	0.9021	0.2592	0.0853	0.2287	0.0499
900	0.8951	0.3243	0.0964	0.3168	0.0787
1,050	0.9177	0.4011	0.1307	0.3749	0.1068
1,200	0.9056	0.4533	0.1572	0.4216	0.1376
1,350	0.9240	0.4814	0.2051	0.4748	0.1940
1,500	0.9132	0.5239	0.2698	0.5152	0.2478

**Table 3.** Comparison of average execution time values for the random search method and the ABC algorithm.

**Number of students**	**Random search method**	**ABC algorithm (number of bees/number of iterations)**			
		20/1,000	20/2,000	100/1,000	100/2,000
150	0.0782	0.2858	0.5515	1.4094	2.7547
300	0.1563	0.6217	1.1781	3.0375	5.8640
450	0.2343	0.9409	1.7969	4.6500	9.0484
600	0.3110	1.3546	2.5218	6.8343	12.4750
750	0.3890	1.8109	3.0844	9.1328	15.1594
900	0.4703	2.2734	3.7375	11.4654	18.5890
1,050	0.5437	2.7811	4.6000	13.9204	22.8953
1,200	0.6219	3.2548	5.4141	16.3031	27.1750
1,350	0.7000	3.7563	6.3578	18.7671	31.8344
1,500	0.7781	4.2156	7.2485	21.1874	36.4359
